# Trust, Sociability, and Quality of Life of Sub-Saharan African Migrants in Germany

**DOI:** 10.3389/fsoc.2021.741971

**Published:** 2021-11-19

**Authors:** Adekunle Adedeji, Tosin Yinka Akintunde, Erhabor S. Idemudia, Elhakim Ibrahim, Franka Metzner

**Affiliations:** ^1^ North-West University, Faculty of Humanities, Mafikeng, South Africa; ^2^ Department of Sociology, School of Public Administration, Hohai University, Nanjing, China; ^3^ Department of Demography, College for Health, Community and Policy, The University of Texas, San Antonio, TX, United States; ^4^ University Medical Center, Hamburg Eppendorf, Department of Medical Psychology, Hamburg, Germany

**Keywords:** trust, sociabilities, quality of life, African migrant, Germany, social capital

## Abstract

Poor social integration is associated with poor quality of life among minority groups. The current study hypothesized that trust and sociability may significantly explain the quality of life performance among Sub-Saharan African migrants in Germany. Data from 518 migrants were analyzed. Hierarchical multiple linear regression models were calculated to assess the predictive effect of trust and sociability on aggregate quality of life. Results show that general trust and sociability explained about 21% of the variance in quality of life score (adjusted *R* ^2^ = .206; *p* < .001) for the total sample. Socioeconomic and demographic features suggested an added predictive effect of about 8% for total sample (adjusted *R* ^2^ = .279; *p* < .001), 10% for male (adjusted *R* ^2^ = .322; *p* < .001) and 4% for female (adjusted *R* ^2^ = .211; *p* < .001). The results support trust and sociability as essential in connecting to a new environment and enhancing the quality of life.

## Background

Migration has become an unavoidable part of human life necessitated by needs and the desire to explore new opportunities to improve income, education, and overall life outcome. When migration occurs, the migrants anticipate a degree of fulfilment of desires and goals enabled by the host country’s socioeconomic, cultural, and environmental dimensions (Kogan et al., 2018). The recent trend in migration creates networks that subsequently evolve into more diverse communities (Manning, 2013). The Sub-Saharan African (SSA) migrants are unique groups whose migration circumstance has been scrutinised in empirical discourse in the last 2 decades (Afulani and Asunka, 2015; Afulani et al., 2016; Zou et al., 2021). Furthermore, while existing literature has highlighted low life satisfaction among SSA migrants (Kogan et al., 2018), the quest to understand this group adaptation and factors that contribute to their well-being remains critical in research and policy intervention.

The quality of life (QoL) assesses the overall subjective evaluation of life (Felce and Perry, 1995). Although QoL is fundamentally individualistic, the research into the scope supports the ever-increasing dynamics of minority people and special groups. The QoL measures dissect individual self-evaluation of essential features of their functioning in diverse spheres of their well-being, encompassing multifacet life experience (Kalpakjian and Tate, 2008). Therefore, investigating the QoL among SSA migrants helps understand their situation and peculiarity to support policy intervention. Numerous literature has streamlined the convolution of migrants’ experiences to expand knowledge on factors affecting their QoL. Essentially, the documentation of the QoL of SSA migrants in China suggested that crucial sociodemographic characteristics, low access to health services, legal and cultural diversity show a significant negative association with QoL (Zou et al., 2021).

In understanding QoL, age and other socioeconomic characteristics have become a vital domain to explore. Age is an essential factor affecting the environmental, physical, social, and general health of migrants (Xing et al., 2013). In a study among female migrant domestic workers in Singapore, age was directly associated with their overall QoL and satisfaction with life (Anjara et al., 2017). Similarly, among SSA migrants in China, socioeconomic characteristics were vital determinants of QoL, suggesting a decrease in the QoL with increasing age (Zou et al., 2021). Therefore, examining the influence of age on the QoL of migrants may facilitate the understanding of other psychosocial factors such as trust and sociability while exploring their unique effects in different geographical settings.

Trust is a multidimensional psychosocial domain, and the theoretical conception is premised on various models cutting across multidisciplinary research (Rousseau et al., 1998; Castelfranchi and Falcone, 2010). However, there is no uniform definition of trust. Therefore, researchers have adopted the idea subjectively based on social science and cognitive science literature (Castelfranchi and Falcone, 2010). Trust intension is based on one party’s willingness to count on another party while having a sense of security irrespective of the outcome (Mcknight and Chervany, 1996). The primary components of trust involve the attributes of the trustor, the trustee, the level of interactions and relationships shared among them, the particular set of actions, and the premise in which trust is conceptualized (Stern and Coleman, 2015). Within the context of this research, trust was conceptualized among migrants based on personal trust toward others (Ann Arbor, 1971), as it helps develop mutual relationships (McAllister et al., 2006).

Although trust has been explored in different contexts, such as social trust (Kim, 2021), generalized trust (Wu, 2021), interpersonal trust (Tokuda et al., 2008), it is increasingly gaining attention as a measure of well-being, especially among migrants. Irrespective of the dimension and the context in which it is measured, trust is perceived as the belief that springs from continual fellowship and cooperation among people in the same environment, and if a positive outcome is derived, it fosters growth and community interaction (Gray, 1997; Chavez et al., 2006). In the human sphere, displaying or receiving trust is a production of the extent to which one recipient is willing to engage in given action with another person accepting the risks and positive outcomes (Seligman, 1998; Goos et al., 2005; Kwantes et al., 2018).

SSA migrant groups face challenges connected with individual experience in the host country. These challenges cause migrants to look inwardly among people who share similar cultural backgrounds to form a network to overcome potential problems in settling and living in a new country (Vidyattama, 2017). Therefore, it is conceptualized that a certain level of trust should be displayed before SSA migrants in Germany can participate in a social network that facilitates their integration. The stronger the level of trust projected by a migrant, the stronger the connection and bond with others. (Chavez et al., 2006). Institutional support can also facilitate trust among migrants. Migrants’ integration programs and policies in the host environment strongly link with trust (Tatarko, 2020). Understanding trust among the SSA migrants and the effect on QoL requires extensive investigation as most evidence on migrants is based on institutional support such as encouraging migrants to participate more in problem-solving (Nahm, 2015), helping migrant families with children (Khalfaoui et al., 2020), and Migrant Integration Policies (Tatarko, 2020). In the case of SSA migrants, moving from their natural habitat to a new territory may envelop their instinctive ability to display trust based on the premise that westernisation may influence their cultural perception of trust (Idemudia and Olawa, 2021).

While sociability is rooted in social capital, it focuses on networking with people with similar attributes to facilitate harmony and cooperation. Sociability is an interaction that fosters collaboration in which one person joins another to perform activities through communication and cooperation (Schiller et al., 2011; Argyle, 2013). Sociability supports how individuals appraise people and groups that help form social judgment (Crocetti et al., 2019). Sociability can also be interpreted as a form of daily interaction (Schiller et al., 2011) supported by interactions with other persons out of shared interest (Simmel, 2015). In the simplest form, sociability is the capacity to interact with other people.

Building on the theory of reciprocity (Falk and Fischbacher, 2006), it is expected that trust and sociability among SSA migrants in Germany will correspond to better integration experience in the host community, facilitate social and environmental interaction, and ultimately improve QoL. However, linkages between sociability, trust, and QoL of SSA migrants are yet to be understood among global migrants. Although sociability and trust are independently studied among minority groups (Hipp et al., 2014; Wu, 2021), they offer extensive exposition on transnationalism and social porosity embedded in the environment. There is currently a lack of evidence on the sociability of SSA migrants and the display of trust to facilitate improved QoL. SSA migrants face significant challenges when embedding and trying to access essential services like healthcare, and jobs in the host countries (Christiaensen et al., 2019; Mbanya et al., 2019). Exploring if SSA migrants in Germany display trust and engage in sociability may offer a promising approach in understanding their QoL and provides templates for policy intervention to better the migration experience of the group.

Thus, this study examined the empirical evidence that trust and sociability (in the form of bridging or bonding social participation) among SSA migrants may significantly be ideal for understanding the QoL of the SSA migrant group in Germany and subsequently support evidence to measure up the experience of other migrants in different settings. However, as evidence is scarce on the influence of trust, sociability on QoL among SSA migrants globally, to our knowledge, this is the first empirical research adopting a quantitative measure to investigate these psychosocial indicators among the migrant groups in Germany. This study, therefore, attempts to extend the empirical evidence on trust, sociability and QoL by using a population of SSA migrants in Germany to:

1. Assess the bivariate relationship between quality of life, trust, sociability, and socioeconomic and demographic features.
2. Analyze the predictive effect of trust and sociability on quality of life.
3. Explore the predictors of quality of life by gender.

## Methods

### Study Design, Population, and Data Collection

Data from 518 SSA migrants in Germany were analyzed. The data were collected in a cross-sectional survey across the 16 German federal states. Participants include SSA migrants from the 49 sub-Saharan countries currently residing in Germany who hold a Germany’s formal residence status (i.e., documented SSA immigrant). Survey questionnaires were completed in German, English, and French. Furthermore, all participants were 18 years or older and lived in one of Germany’s 16 federal states.

A standardized version of each adapted measure in German, English, and French was used. No translation was, therefore, necessary. Of the 532 total participants, 373 completed the online questionnaire, and 95 filled out the paper and pencil survey. In contrast, the questionnaire was administered to the remaining 64 one-on-one or via telephone interview. Data from 14 participants were removed due to missing data. The Study sample was reached using the 5-wave-approach—developed to address the non-representativeness in the Sudman and Kalton snowballing sampling technique. This approach breaks down the discriminative referral system into separate components that systematically increase the probability of selection for all population members (Adedeji, 2019).

### Measure

#### Quality of Life

QoL was quantified using the EUROHIS-QOL 8-item Index (Schmidt et al., 2006). This self-report questionnaire is derived from the World Health Organization Quality of Life Assessment (WHOQOL-100 and WHOQOL-Bref instruments) (WHOQOL, 1998). It includes eight items covering: subjective quality of life, satisfaction with health, energy and fatigue, financial resources, satisfaction with sleep and rest, self-esteem, personal relationship and home environment. Each item was scored on a 5-point Likert scale ranging, for instance, from “not at all” to “completely.” An overall QoL score was computed from the mean score of the items ranging from 8 to 40, with higher scores indicating better QoL. The score was then categorized based on the 5-point Likert scale for descriptive analysis. The questionnaire presented good reliability in the current sample, with a Cronbach’s alpha value of .83 (Blanz, 2015).

#### Trust

Trust was measured using a 3-item scale designed to measure individuals’ general level of trust toward other people (Ann Arbor, 1971). The three items were first used in the 1964 post-election study conducted by the Survey Research Center and have continued to be used in national surveys. These items have been established as a valid measure of trust in the social context (Yamagishi, 1986; Hetherington, 1998). Each of the three items was coded into dichotomous choices. One of the two choices is the high trust response, and the other is considered the low trust response. For example, the first item: “Generally speaking, would you say that most people can be trusted or that you cannot be too careful in dealing with people?” 1 = Most people can be trusted; 0 = can’t be too careful. A total trust was computed as the sum score for the three items, ranging from 0 to 3. A 0 score means very low trust, while a score of 3 implies a very strong level of trust.

#### Sociability

Two sociability variables were conceptualized based on the definition as the capacity to interact with other people. Firstly, sociability as a form of social participation was measured using a sub-scale of Grootaert and Bastelar, (2002) integrated measure of social capital. The numbers of social meetings with people in public places, their homes or at participants’ own homes, and participation in community events such as sports or ceremonies were recorded as a continuous variable to measure sociability—social participation. A second item was used to distinguish whether sociability is of the bonding (maintaining existing social relationship) or bridging (creating new social relationship) variety. Participants were asked using dichotomous choice whether the people with whom they meet are of the same or a different ethnic or linguistic group, economic status, social status, or religious group (1 = different; 2 = the same). A high score (8 or 7) suggest bonding sociability, while a lower score (4 or 5) indicates bridging sociability. A score of 6 is coded as bonding and bridging sociability.

#### Socioeconomic and Demographic Measures

Participants’ age in years, gender (male = 1; female = 2), marital status, education attainment, income, and education, were collected. Participant education attainment was measured as the highest completed education or vocational training. On the other hand, income was measured as respondents’ approximate gross annual income with options from less than 10,000 Euro to above 50,000 Euro a year. This categorization is intended for a more straightforward classification of participants into income groups based on Germany’s individual income tax (BZSt - Tax return, 2020). Similarly, participants were asked to evaluate their current occupation based on education completed (BAMF - Bundesamt für Migration und Flüchtlinge, 2005).

#### Data Analysis

Descriptive statistics were computed for sociodemographic, socioeconomic, trust, sociability, and QoL variables. A correlation matrix exploring the bivariate associations between SSA migrants’ QoL, trust, sociability, socioeconomic and sociodemographic features were computed. Correlation coefficients were interpreted as small (*r* = .10), medium (*r* = .30) or large (*r* = .50) (Cohen, 2013). Hierarchical multiple linear regression models were calculated to assess the predictive effect of trust, sociability (social participation and variety—bonding or bridging) on QoL. Model 1 explores the predictive effect of—trust and sociability on quality of life. Model 2 explores the added predictive effect of sociodemographic and socioeconomic features to model 1.

Effect sizes and *p*-values are reported for the regression model. The overall fit of the models was evaluated by adjusted *R* ^2^ statistics (Nagelkerke, 1991); R-Change and F-test determined the significance of changes in model fit. To interpret the regression coefficients of the regression models (*β*), we used guidelines by Cohen (2013): *β* = .1 indicated a small, *β* = .3 a medium, and *β* = .5 a large effect. The significance level was determined as *p* < .05 for all analyses. Analyses were computed using IBM SPSS Version 26.

## Results

### Sample Description

Data from SSA migrants (*N* = 518) were included in the current analysis. Data on age shows that ”participants’ age ranges between 19 and 56 years, with an average age of 32.5 years (SD 7.93). As shown in Table 1, the majority (61%) of the study participants were male. A little over 60% reported being single or in an unmarried partnership as marital status. Analyses of data on socioeconomic status show that about 60% of the participants earn below 20,000 Euro per annual. On the other hand, half of SSA migrants had completed at least a university degree. At the same time, about 60% were occupied below their qualification level.

**TABLE 1:** Socioeconomic and demographic characteristics of participants (*N* = 518).

Characteristic	n	%
Gender
Male	315	60.8
Female	203	39.2
Marital Status
Single	182	35.1
Widowed	2	0.4
Divorced	8	1.5
In partnership, but not married	158	30.5
Married	168	32.4
Highest education level
None	6	1.2
Secondary education or elementary school certificate	88	17.0
Vocational school certificate	164	31.7
Degree from a university	172	33.2
Master, technician, or equivalent certificate	88	17.0
Length of stay
Less than 1 year	18	3.5
1–3 years	77	14.9
3–5 years	79	15.3
5–10 years	115	22.2
More than 10 years	229	44.2
Occupation
I am not exercising an occupation at present	18	3.5
I am occupied below my qualification level	305	58.9
I am occupied at my qualification level	195	37.6

### Trust

The sample trust score ranged between 0 and 3. Descriptive analysis returned a mean score of 0.9 (SD 0.87) for the total sample. Thus, about 40% reported zero (no) trust; another one-third of the total sample show low trust, about 24% have moderate trust in people. Only about 3% have high trust in other people.

**FIGURE 1:**
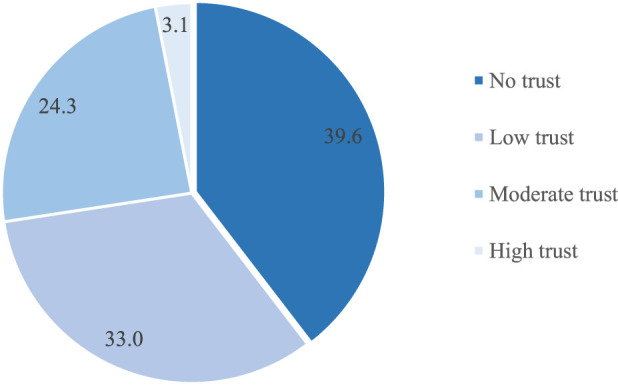
Percentage distribution of trust score among SSA migrants in Germany (*N* = 518).

Gender-specific analysis shows statistically significant difference in the trust scores for males (*M* = 0.98, *SD* = 0.87), and for females (*M* = 0.79, *SD* = 0.86); *t* (516) = −2.45, *p* = 0.014. Item-specific analysis shows that only about 35% of the participants reported that most people could be trusted. Consequently, less than half (45%) believe that people try to be helpful most of the time, while about 90% think most people would try to take advantage of them if they got the chance (see Figure 1).

### Quality of Life

Analysis of aggregate QoL reveals that SSA migrants reported an average score of 3.4 (SD 0.7) with a minimum score of 1.5 and a maximum score of 4.75. Gender-specific analysis shows significant difference in the QoL mean score for males (*M* = 3.47, *SD* = 0.69), and for females (*M* = 3.34, *SD* = 0.64) condition; *t* (516) = −2.11, *p* = 0.035. Comparison of SSA migrant QoL mean score to the Germany norm population score reported by Schmidt et al. (2006) shows a poorer QoL for the total SSA male and female participants. Table 2

**TABLE 2:** Descriptive and psychometric properties for the total quality of life score.

	Sample	Α	Total		Male		Female		t	Sig. (2-tailed)
			Mean	SD	Mean	SD	Mean	SD		
Germany Norm^a^	401	0.80	4.08	0.49	4.03	0.52	4.14	0.44		
SSA Migrants	518	0.83	3.40	0.67	3.47	0.69	3.34	0.64	−2.14	.03

a Germany EUROHIS-QOL norm score as presented by Schmidt et al., 2006.

### Sociability

**FIGURE 2:**
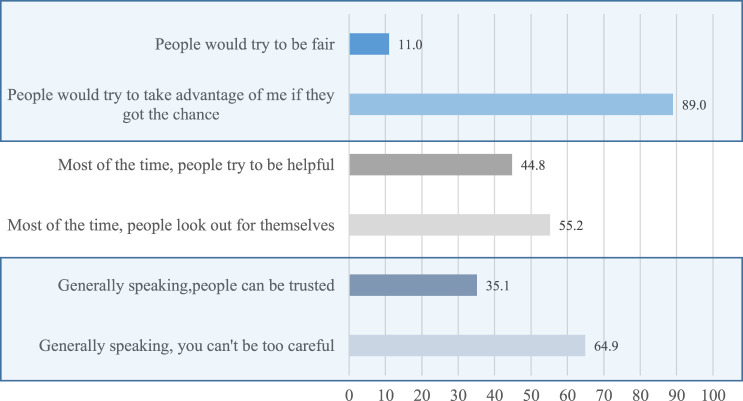
Descriptive distribution of trust among SSA migrants in Germany (*N* = 518).

**FIGURE 3:**
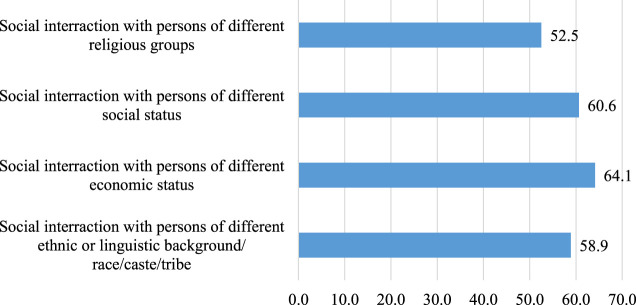
Percentage distribution of bridging social interactions among SSA migrants in Germany (*N* = 518).

**FIGURE 4:**
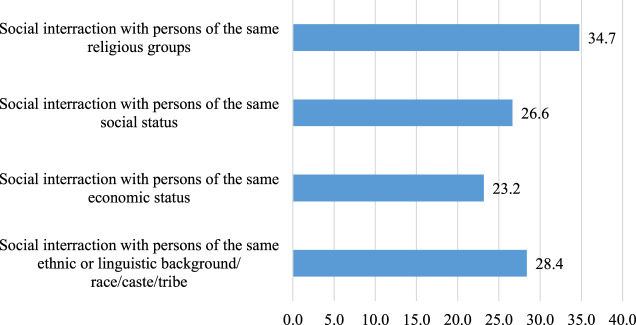
Percentage distribution of bonding social interactions among SSA migrants in Germany (*N* = 518).

The frequency of sociability (social participation) shows SSA migrants in the current sample reported meeting with people to have food or drinks, either in their home or in a public place, on average, about three times during the past 4 years weeks. The reported frequency, however, ranged from 0 to 10 times. Approximately 66 (12%) participants reported zero sociability, while about 30% reported more than three social interactions. Further analysis to distinguish whether the reported sociability was of the bonding or bridging variety shows 61.5% of reported sociability were of bridging combination (with diversity score 4 or 5). Another 20.4% were of bonding (diversity score 7 and 8). In comparison, the remaining 18.1% were both bonding and bridging varieties (diversity score 6). As shown in Figures 2–4 below, item-specific analysis shows that participants’ sociability was most diverse in terms of economic status and least diverse in terms of religious affiliation.

### Analysis: Correlation Matrix

A’ ‘Pearson’s correlation coefficient matrix was computed to examine the association between quality of life, trust, sociability, socioeconomic (educational attainment, occupation, income), and demographic (age and length of stay in Germany) features. As presented in Table 3 below, participants’ QoL shows significant association with trust (*r* = .42; *p* < 0.01), social participation (*r* = 0.29; *p* < 0.01), and whether these interactions are of bridging or bonding variety (*r* = −0.27; *p* < 0.01). Similarly, education (*r* = 0.34; *p* < 0.01) and occupation (*r* = 0.10; *p* < 0.05) returned a positive correlation with the QoL while age (*r* = −0.12; *p* < 0.01) and length of stay (*r* = −0.09; *p* < 0.05) were negatively associated with participants QoL. Income, however, did not correlate with the QoL but showed significant results with trust (*r* = 0.12; *p* < 0.01) as well as, for sociability—social participation (*r* = −0.15; *p* < 0.01), and whether these interactions are of bridging or bonding variety (*r* = 0.10; *p*< 0.05).

**TABLE 3:** Correlation matrix (*N* = 518).

		Quality of life	Trust	Sociability		Education attainment	Income	Age	Length of stay in Germany
				Social participation	Bridging—bonding				
Quality of life		1							
Trust		0.421**	1						
Sociability	social participation	0.291**	0.091*	1					
	Bridging—Bonding	−0.271**	−.024	−0.252**	1				
Education Attainment		0.338**	0.146**	0.157**	−0.020	1			
Income		0.012	0.117**	−0.148**	0.098*	−0.016	1		
Age		−0.215**	0.020	−0.295**	0.234**	−0.041	0.361**	1	
Length of stay		−0.090*	0.030	0.035	0.021	−0.102*	0.303**	0.143^**^	1

Boldface, significant; **p < 0.01 (2-tailed); *p < 0.05 (2-tailed).

### Regression Model

The multiple linear regression model (Model 1) shows a statistically significant association between the dependent variable, QoL, and the predictors, trust and sociability (adjusted *R* ^2^ = 0.206; *p* < 0.001) for the total sample. Similarly, results from the gender-specific analysis suggest that trust and sociability account for 24% variance in male QoL scores and 19% for females. Model 2, assessing the added effect of socioeconomic and demographic features, suggested an added predictive effect of about 8% for total sample (adjusted *R* ^2^ = 0.279; *p* < 0.001), 10% for male (adjusted *R* ^2^ = 0.322; *p* < 0.001) and 4% for female (adjusted *R* ^2^ = 0.211; *p* < 0.001) participants. The change in effect size from Model 1 to Model 2 was significant for the total sample of males but not significant for the female participants (Change in F = 1.948, *p* = 0.089) Table 4.

**TABLE 4:** Hierarchical multiple regression models exploring trust, sociability, and quality of life among SSA migrants in Germany.

	*B*	*β*	*P*	*Adjusted R* ^*2*^	Δ*f*	*p*Δ*F*
		**Model 1**				
**Total (*N* = 518)**				0.206	39.969	0.001
*Constant*	3.760*		0.000			
Trust	0.250*	0.339	0.000			
Social Participation	0.039*	0.127	0.004			
Bridging and Bonding	−0.117*	−0.231	0.000			
**Male(*N* = 315)**				0.238	29.417	0.001
*Constant*	4.104*		0.000			
Trust	0.216*	0.274	0.000			
Social Participation	0.044*	0.123	0.026			
Bridging and Bonding	−0.172*	−0.330	0.000			
**Female(*N* = 203)**				0.190	14.834	0.001
*Constant*	3.214*		0.000			
Trust	0.264*	0.399	0.000			
Social Participation	0.045*	0.176	0.013			
Bridging and Bonding	−0.027	−0.055	0.433			
		**Model 2**				
**Total (*N* = 518)**				0.279	22.865	0.001
*Constant*	3.488*		0.000			
Trust	0.252*	0.342	0.000			
Social Participation	0.025	0.082	0.064			
Bridging and Bonding	−0.100*	−0.197	0.000			
Education	0.136*	0.216	0.000			
Income	0.008	0.020	0.674			
Age	−0.010*	0.004	0.003			
Length of stay	−0.049*	0.022	0.025			
**Male(*N* = 315)**				0.322	17.171	0.001
*Constant*	3.921*		0.000			
Trust	0.239*	0.303	0.000			
Social Participation	0.017	0.000	0.386			
Bridging and Bonding	-0.149*	−0.286	0.000			
Education	0.139*	0.215	0.000			
Income	0.009	0.019	0.747			
Age	−0.013*	−0.155	0.006			
Length of stay	−0.062*	−0.122	0.025			
**Female(*N* = 203)**				0.211	6.932	0.089
*Constant*	2.933*		0.000			
Trust	0.260*	0.392	0.000			
Social Participation	0.034	0.134	0.080			
Bridging and Bonding	−0.020	−0.040	0.574			
Education	0.121	0.183	0.080			
Income	0.009	0.022	0.781			
Age	−0.007	−0.098	0.227			
Length of stay	−0.017	−0.029	0.686			

The results from Model 1 also support trust as a significant predictor for QoL for the total sample (*β* = 0.339; *p* < 0.001) as well as for male (*β* = 0.274; *p* < 0.001) and female (*β* = 0.399; *p* < 0.001) participants separately. While the frequency of social participation was also significant for both genders and the total sample, the variety of social interactions in bridging or bonding was not significant for the female QoL score.

After adding socioeconomic and demographic features in Model 2, trust maintained its significance for QoL for the total sample (*β* = 0.252; *p* < .001), for male (*β* = 0.239; *p* < 0.001) as well as for female (*β* = 0.260; *p* < 0.001). On the other hand, the frequency of social participation was not significant; however, whether these interactions were of bridging or bonding variety was significant for the total sample (*β* = −0.197; *p* < 0.001) and for the male participants (*β* = −0.286; *p* < 0.001). Following Cohen (2013), while education and occupation have a unique, significant, positive contribution to Model 2, predicting participants’ QoL, age, and length of stay in Germany inverse contribution to the model. A gender-specific analysis shows no unique contribution from the socioeconomic and demographic features for females. For males, however, education, age, and length of stay in Germany have a unique contribution to Model 2 predicting sample QoL.

## Discussion

This study explores trust, sociability, and QoL among SSA migrants in Germany. Addressing the significant shortage of empirical evidence on trust and the sociability of SSA migrants globally, the current research explores the predicting effect of trust, and sociability on the QoL of SSA migrants in Germany. The research focal point was to emphasize the connections between the QoL, trust, sociability, and sociodemographic characteristics of SSA migrants. Furthermore, there has been particular attention on these groups with studies exploring the migration experience of SSA migrants, such as subjective integration, cognitive and social capital on their well-being (Adedeji and Bullinger, 2019; Adedeji et al., 2019).

The average QoL score for SSA migrants projects a lower score for SSA migrants than the norm population in Germany (Schmidt et al., 2006). This result emphasises the challenges faced by SSA migrants in Germany. The poor integration in their new socio-cultural and economic environment and limited access to infrastructure that facilitates positive life outcomes are arguably responsible for the lower score.

The correlation explored allowed for the comparison of association across the study domains. Trust and sociability resulting from social participation were positively associated with the QoL of SSA migrants in Germany. Among the SSA migrants, engaging in social activities such as social meetings and community events is essential for improving their QoL. Similarly, the sociability premised from bonding to bridging shows bridging sociability associate with sample higher QoL score. These results complement other research where social participation was identified as a predictor of QoL (Arpino and de Valk, 2018; Carver et al., 2018; Guo et al., 2018; Santini et al., 2020). Similar to the findings from the current study, analyses of representative data from 28,982 adults from 12 European countries also suggest participation in formal social functions was a protective factor against the onset or development of chronic conditions, enhanced quality of life, and diminished depressive symptoms (Santini et al., 2020). For SSA migrants in Germany, this association between sociability and QoL can be linked with the indirect effect of integration. SSA migrants with higher bridging social participation are arguably more exposed to socioeconomic and environmental factors that facilitate quality of life (Hoebel et al., 2017; Wüstemann et al., 2017).

Similarly, higher trust among the SSA migrants is instrumental in achieving optimal QoL, especially among women. This result was supported by Salehi et al. (2015), where trust was associated with women’s QoL through facilitating participation and reciprocity at the level of individuals and informal social groups. Trust arguably increases access and usage of infrastructure that corresponds to or promotes improved QoLamong SSA migrants in Germany (Tokuda et al., 2008).

Additionally, the socioeconomic status of the SSA migrants, such as educational attainment, contributes significantly to their QoL. This evidence supports the assumption that advancement in education corresponds with an improved QoL. On the other hand, SSA migrant attributes such as age and length of stay in Germany are negatively associated with QoL. This evidence contrasts with the study in South Africa, where migrants who have stayed for a more extended period reported improved life satisfaction and economic well-being (Chikowore and Willemse, 2017). The contrasting empirical evidence may be ascribed to the socioeconomic and environmental diversity between South Africa and Germany and the experience and integration of SSA migrants. However, similar results were found in another study where older migrants are more likely to be less satisfied with their lives in the host country (Kogan et al., 2018).

Sociability regarding social participation among the SSA migrants holds a significant association in establishing trust among the SSA migrant population. This agrees with Chavez et al., where trust was argued as a foundational concept for understanding sociability (Chavez et al., 2006). Similarly, income and education attainment were two socioeconomic attributes that support and facilitate trust among the migrant group. These help the understanding that migrants with higher income and education show higher trust. Sociability in terms of social participation and sociability (bridging—bonding) were inversely associated, suggesting that higher social participation corresponds to more bridging sociability. Only education attainment improves the social involvement of the migrants. When the age and income of SSA migrants increase, there is a reduced social participation among the group. For bridging and bonding, increasing revenue and age encourages bonding of existing relationships.

Existing evidence identified the potential of reduced trust among Africans due to ethnic diversity, as SSA migrants in Germany are products of different countries in Africa (Idemudia and Olawa, 2021). Trust and social participation among the SSA migrant in Germany supports the understanding of QoL. Increased trust and social participation improves participant’s QoL outcomes. However, the net effect of trust and sociability (social participation and bridging/bonding) among SSA migrants projects approximately 21% of their QoL. This result validates a study that established that trust is positively associated with a better QoL (Tokuda et al., 2008). Bridging—bonding evidenced in the outcome point towards the bridging sociability as a predictor of QoL among the study sample.

A contrasting experience based on trust, social participation, and bridging–bonding between males and females also impacts SSA migrants’ QoL. For male SSA migrants, trust, sociability significantly explained the variance in their QoL. Trust and social interaction and bridging sociability were fundamental in improving the QoL. Female SSA migrants’ QoL were positively influenced by trust and social participation. This evidence project higher social participation and trust enjoyed by SSA male migrants in Germany than female SSA migrants. Thus, other factors may explain the insignificance of bridging and bonding in explaining the QoL of female SSA migrants in Germany. Similarly, female migrants are more positively impacted by trust compared to male migrants, and this could be true of trust, as the degree of trust displayed among people of different cultural backgrounds may vary (Wu, 2021).

The overall QoL of SSA migrants was improved by including socioeconomic and demographic characteristics such as educational attainment of SSA migrants in Germany, which provides additional support towards their QoL. In agreement with findings from other studies, education offers favourable observable characteristics to better the QoL among migrants (Ohaeri and Awadalla, 2009; Adedeji, 2019). The more opportunities explored in education, the greater their overall QoL. In contrast, the age factor and the length of residency in Germany show a detrimental effect on the QoL of SSA migrants. The increase in age and length of stay of SSA African migrants suggest a dwindling QoL. The aggregate impact of trust, sociability, education, age, and duration of residency explains approximately 28% of the variance in the QoL of SSA in Germany.

Gender analysis presents different results for male and female SSA migrants. The current investigation offers male SSA migrants’ QoL as products of trust, bridging sociability, education, age, and length of stay in Germany, which explains 32% of their QoL. However, for male SSA migrants’, age and duration of stay in Germany negatively influence the QoL. This is in contrast to evidence that living in a place for an extended period supports community interaction (Ramos et al., 2017). The traditional backgrounds of SSA may be accountable for the disparity in the experience of male and female migrants’ QoL in Germany based on the noncontributing factors of income, education, and length of stay in Germany for female SSA migrants. In Africa, the traditional societies are primarily patriarchal (Nwagbara, 2020). The males are considered the primary provider in the family and limit females’ economic and education potentials. This historical setup of African female migrants may transcend into their experience living in foreign countries, which may have impacted the overall benefit of education and income.

## Limitation

Like any empirical research, this study has some limitations when interpreting and adapting the research findings. The study participants’ recruitments appraised only registered migrants who responded to survey invitations. Thus, the sample size may not account for the totality of SSA migrants in Germany. Furthermore, the cross-sectional nature of the analysis limits the interpretation of the result. A more comprehensive study that allows participation from SSA migrants regardless of their status and operationalised QoL measures and its predictor will provide more explicit information on QoL determinants for the SSA migrants group.

## Conclusion

The evidence from this study supports the effect of trust and sociability on the QoL of SSA migrants in Germany and establishes trust as a crucial factor in integrating and enhancing QoL. The peculiarity of female SSA migrants warrants further research to understand other psychosocial functions that may improve their QoL. Generally, the current investigation results encourage building psychosocial support that may improve trust and facilitate sociability among people of ethnic diversity and transcend into improved QoL. While the research findings may have presented the sociability, length of stay in Germany, socioeconomic and demographics characteristics as unattributed to female SSA migrants’ QoL, it encourages investigating salient factors peculiar to this group to understand their experiences and QoL.

## Data Availability

The raw data supporting the conclusion of this article will be made available by the authors, without undue reservation.

## Abbreviations

QOL, Quality of life; SSA, Sub-Saharan African; WHO, World Health Organization.
